# Supplementary material for: Micron-resolution fiber mapping in histology independent of sample preparation
Source: Nat Commun. 2025 Nov 5;16:9572. doi: 10.1038/s41467-025-64896-9 (PMC12589536; doi:10.1038/s41467-025-64896-9)
Supplement: Supplementary file 1 — Supplementary Information [file 41467_2025_64896_MOESM1_ESM.pdf]

Micron-resolution fiber mapping in histology independent of sample preparation

Marios Georgiadis[1✉], Franca auf der Heiden[2], Hamed Abbasi[3,4], Loes Ettema[3], Jeffrey Nirschl[5], Hossein Moein Taghavi[1], Moe Wakatsuki[1], Andy Liu[1], William Hai Dang Ho[1], Mackenzie Carlson[1,6], Michail Doukas[7], Sjors A. Koppes[7], Stijn Keereweer[4], Raymond A. Sobel[5], Kawin Setsompop[1], Congyu Liao[1], Katrin Amunts[2,8], Markus Axer[2,9], Michael Zeineh[1], Miriam Menzel[3,2✉]

[1]Department of Radiology, Stanford University, Stanford, CA 94305, USA.
[2]Institute of Neuroscience and Medicine (INM-1), Forschungszentrum Jülich GmbH, Jülich, Germany.
[3]Department of Imaging Physics, Faculty of Applied Sciences, Delft University of Technology, Delft, the Netherlands.
[4]Department of Otorhinolaryngology and Head and Neck Surgery, Erasmus MC, University Medical Center Rotterdam, Rotterdam, the Netherlands.
[5]Department of Pathology, Stanford University, Stanford, CA 94305, USA.
[6]Department of Neurology and Neurological Sciences, Stanford University, Stanford, CA 94305, USA.
[7]Department of Pathology, Erasmus MC, University Medical Center Rotterdam, Rotterdam, the Netherlands.
[8]C. and O. Vogt Institute for Brain Research, University Hospital Düsseldorf, Medical Faculty, University Düsseldorf, Germany.
[9]Department of Physics, School of Mathematics and Natural Sciences, University of Wuppertal, Wuppertal, Germany.

Correspondence and requests should be addressed to Marios Georgiadis (email: mariosg@stanford.edu) and Miriam Menzel (email: m.menzel@tudelft.nl).

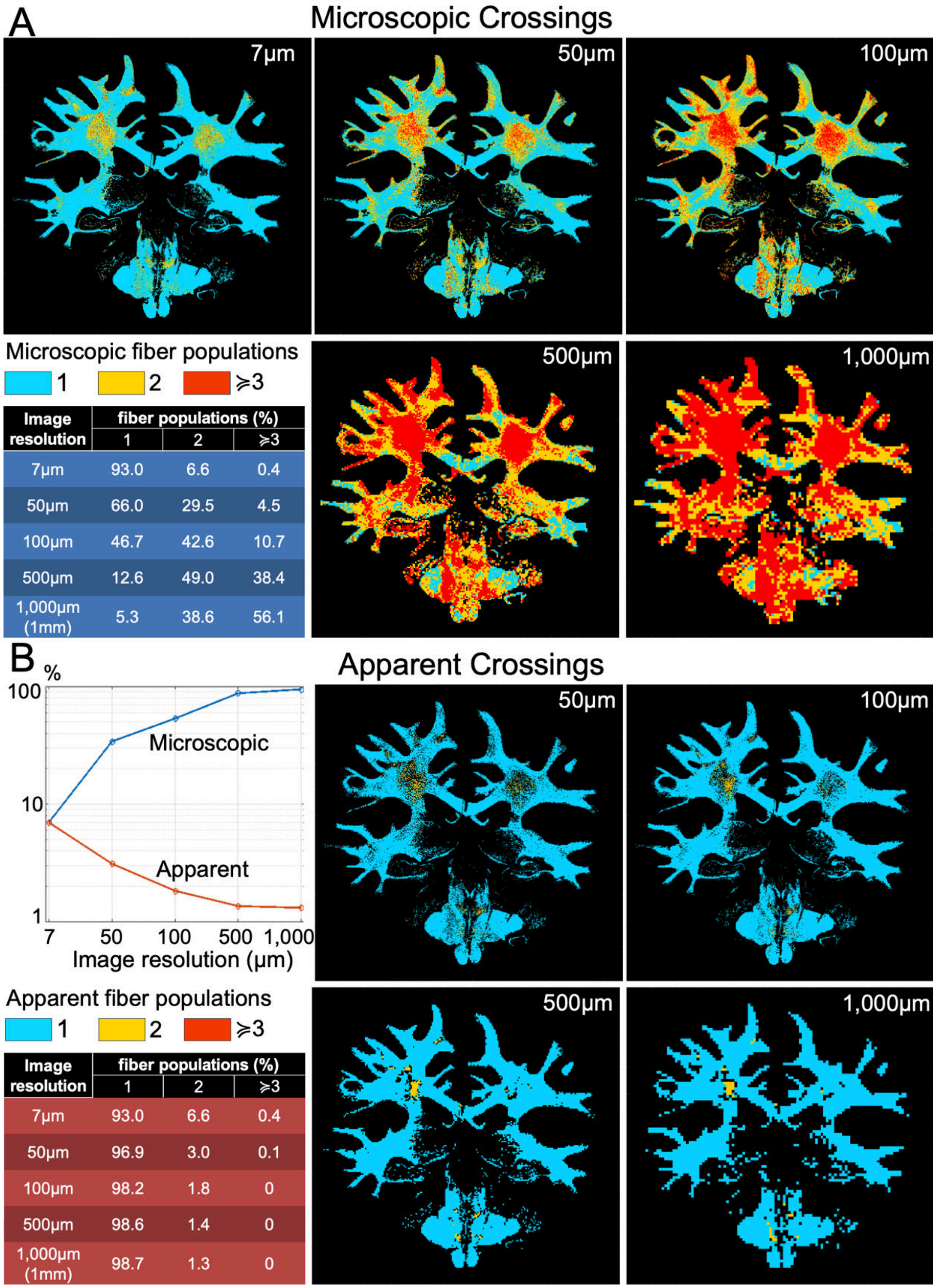

Image resolution	fiber populations (%)		
	1	2	⩾3
7µm	93.0	6.6	0.4
50µm	66.0	29.5	4.5
100µm	46.7	42.6	10.7
500µm	12.6	49.0	38.4
1,000µm (1mm)	5.3	38.6	56.1

Image resolution	fiber populations (%)		
	1	2	⩾3
7µm	93.0	6.6	0.4
50µm	96.9	3.0	0.1
100µm	98.2	1.8	0
500µm	98.6	1.4	0
1,000µm (1mm)	98.7	1.3	0

Supplementary Fig. 1 | Number of fiber populations at different pixel sizes. (A) Presence of microscopic crossings. Maps of the number of microscopic fiber populations with different orientations per pixel, for various pixel sizes, starting from single pixels at the ComSLI resolution for this experiment (7µm), then pixel kernels of 7x7 microscopic pixels (~50µm), 14x14 pixels (~100µm), 71x71 pixels (~500µm), and 143x143 pixels (~1mm). The lower resolution 50µm-1mm pixels were marked as having multiple fiber populations if their kernel contained microscopic (7µm) pixels with multiple fiber populations. **(B) Detectability of crossings by resolution**. For apparent crossings, the original ComSLI image data were downsampled to the respective resolution, and SLIX was used to identify fiber orientations/crossings.

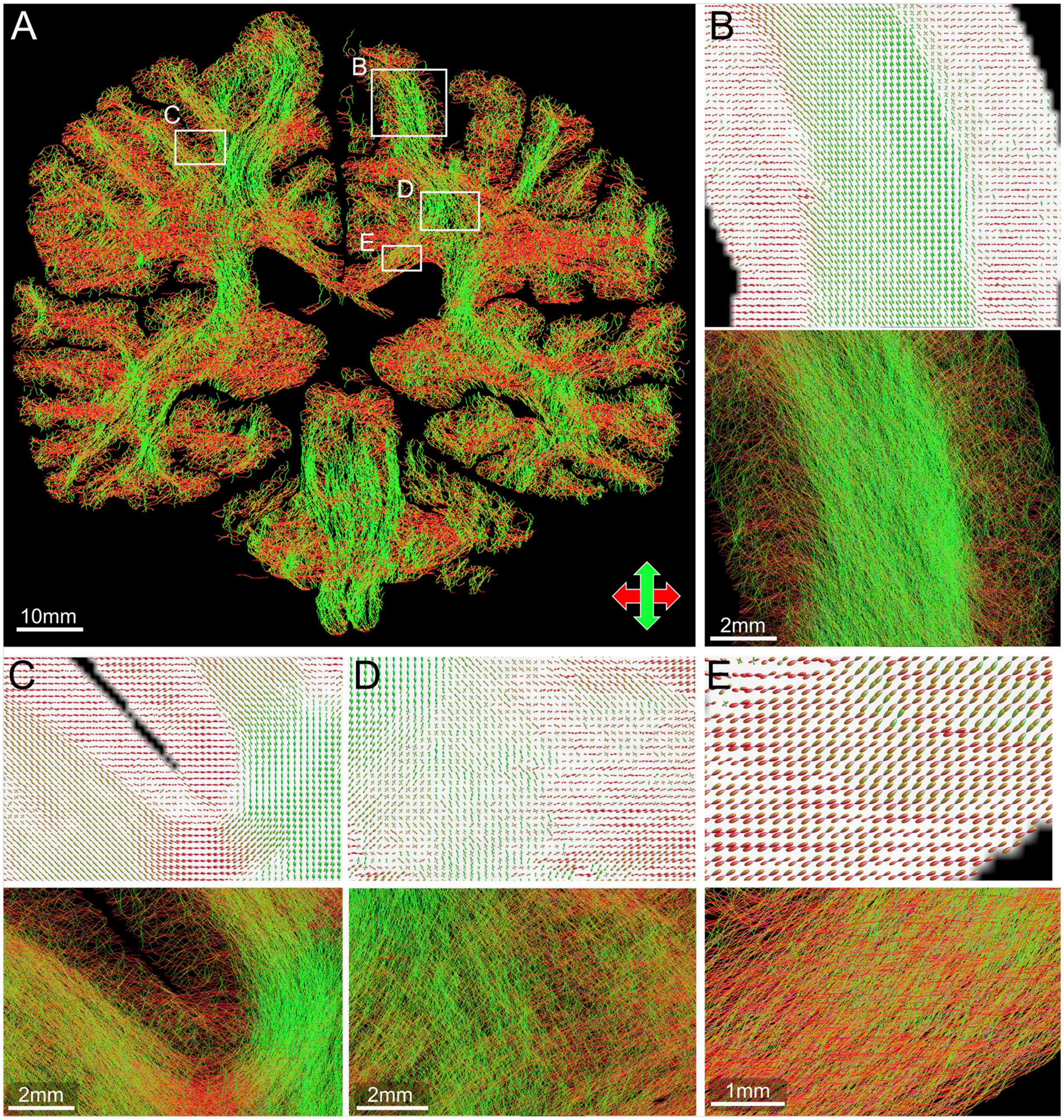

Supplementary Fig. 2 | Microstructure-based orientation distribution functions (ODFs) and whole-brain tractography. (**A**) Tractogram of the whole brain based on ComSLI microscopic fiber orientations for section no. 3452 from the second *BigBrain* dataset, FFPE, silver-stained (same as in main text Fig. 1). (**B-E**) ODFs and tracts for the boxes marked in A. (**B**) ODFs and tracts for a white and gray matter region of the precentral gyrus, where very dense fiber tracts dominate the white matter reflecting the strong, almost unidirectional ODFs, with abundant fibers in the gray matter areas too, mostly following the cortical columns, with some also running across them. (**C**) ODFs and tracts for a white and gray matter region including pre/post-central gyrus U-fibers, with some fibers also connecting into gray matter. (**D**) ODFs and tracts for a part of the corona radiata, with hundreds of fibers seen crossing. (**E**) ODFs and tracts for a part of the corpus callosum, with mostly unidirectional ODFs resulting in fibers running superolaterally. ODFs correspond to microstructure-based fiber orientation distributions (μFODs) of 30×30 pixels (210×210μm^2).

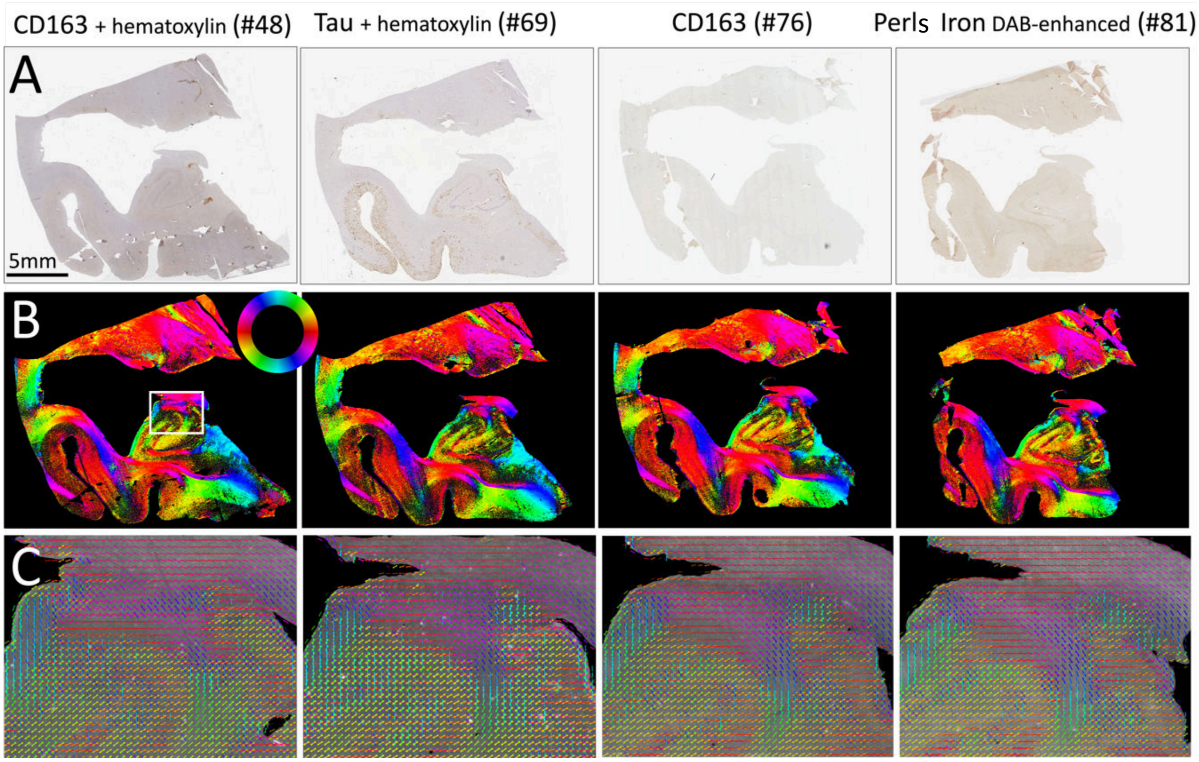

Supplementary Fig. 3 | Differently stained FFPE sections of a human hippocampus measured with ComSLI (same block as in main Fig. 2A-C). (**A**) Brightfield microscopy images, 0.5µm/pixel. (**B**) Color-coded fiber orientation maps from ComSLI, 7µm/pixel. (**C**) Zoomed-in fiber orientation vectors (rectangular region marked by white box in (B)). Vectors of kernels of 15x15 pixels are overlaid for visual clarity.

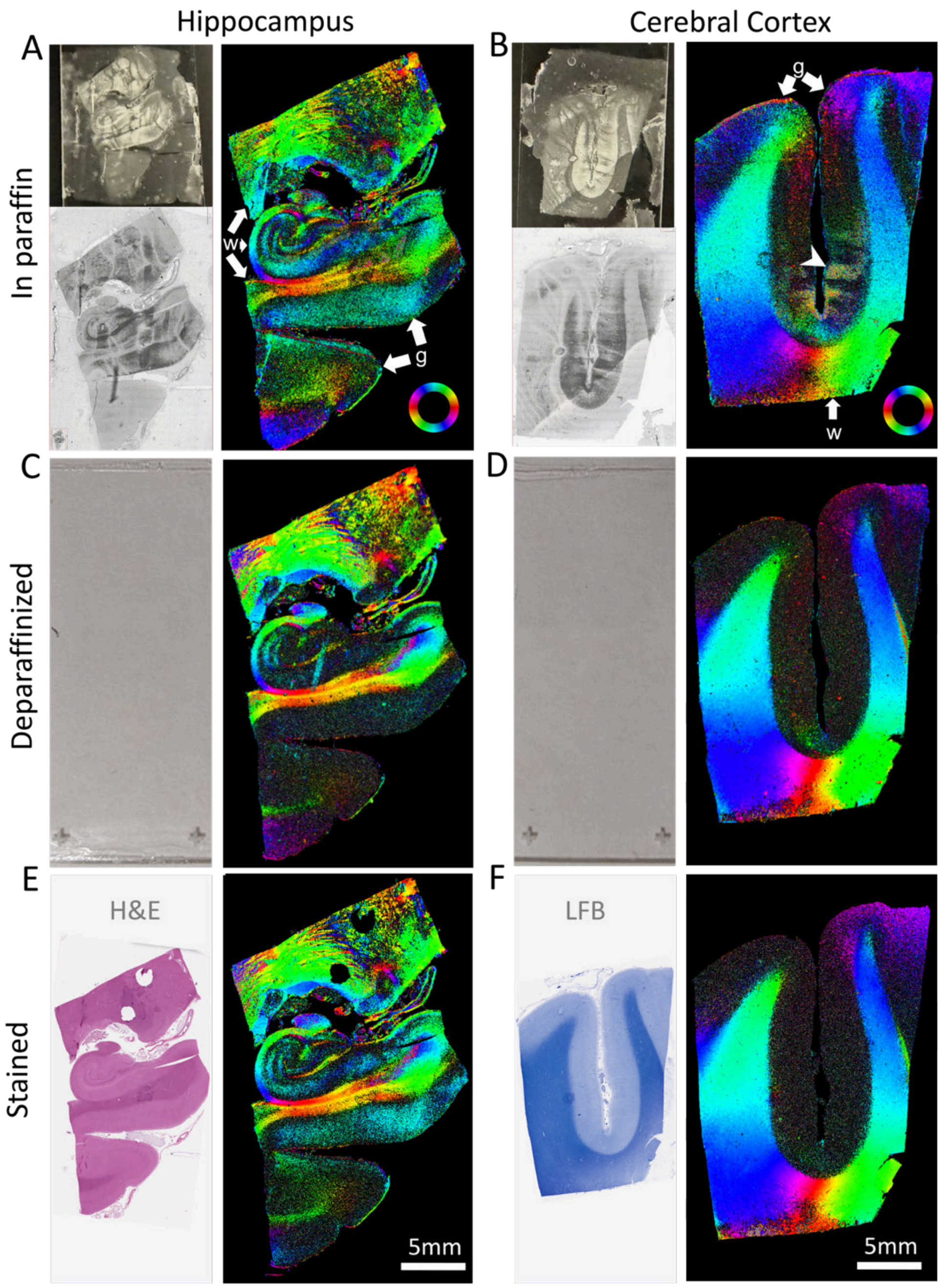

Supplementary Fig. 4 | ComSLI at different steps of FFPE sample preparation. Left: human hippocampus, right: human isocortex and subcortical white matter. Each panel contains brightfield images, 0.5µm/pixel (left side) and the respective ComSLI color-coded fiber orientations, 8µm/pixel (right side). (**A,B**) Sections still in paraffin. The fiber orientations are clearly visible in white (w) and gray (g) matter, despite evident paraffin structures in the photographs (top left corner) and slide-scanned images (bottom left corner). Small folds in the physical section create small artifacts in the gray matter (arrowhead in (B)). (**C,D**) The same sections after deparaffinization, before staining. The sections lack brightfield contrast for slide-scanning so only photographs are shown (left). Retrieved fiber orientations are similar to those in (A,B), especially in the white matter. (**E,F**) The same sections after staining (hippocampus: hematoxylin and eosin (H&E), cortex: Luxol fast blue (LFB)), where orientations follow the same orientation patterns. Overall, gray matter orientations are more pronounced in paraffinized compared to unstained sections. In the hippocampus, gray matter orientations were pronounced in the stained section as well, similar to the hippocampi in main Fig. 2 and Supplementary Fig. 3, owing to the multiple tracts crossing the hippocampal gray matter[1].

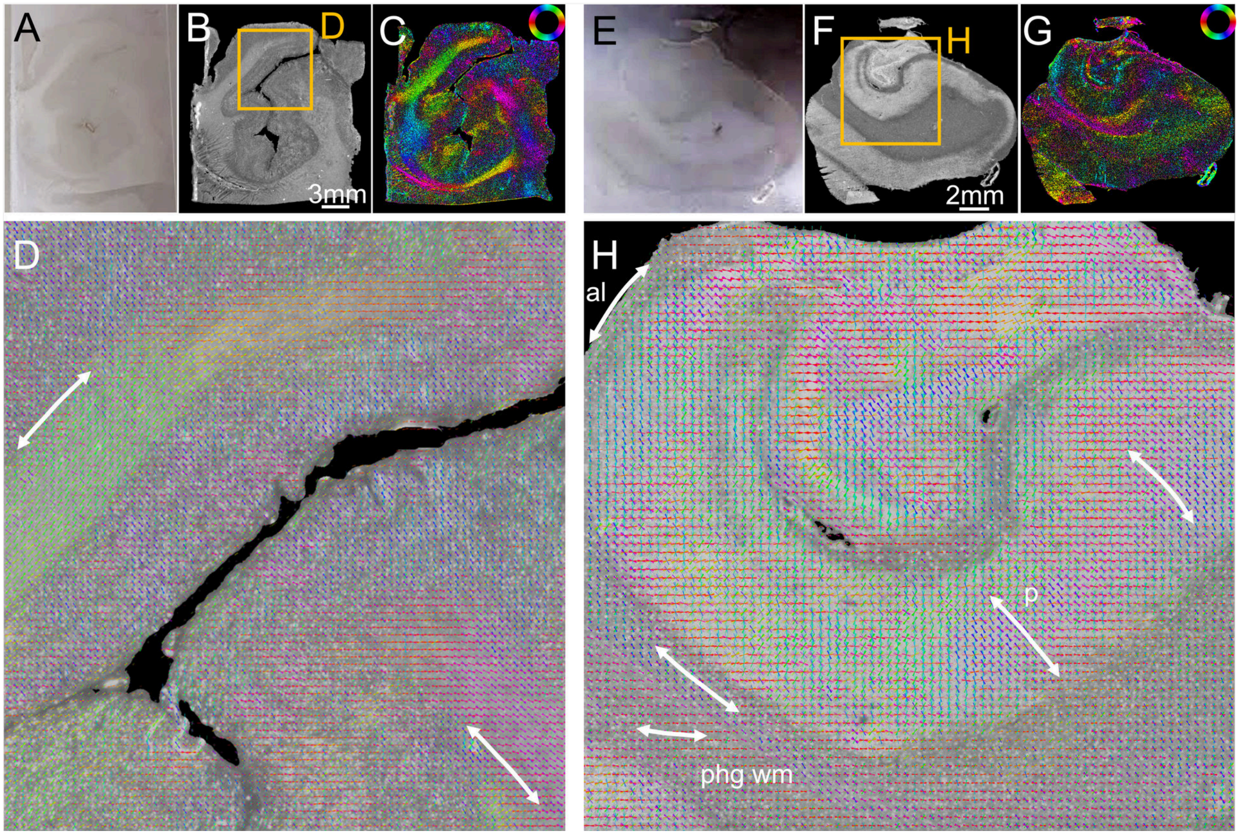

Supplementary Fig. 5 | ComSLI on uncoverslipped fresh-frozen brain tissue sections. (**A**) Human visual cortex, photograph of the frozen section. (**B**) ComSLI average scattering map, 8μm/pixel. (**C**) Corresponding fiber orientation map showing the fiber orientations for each pixel in different colors according to the color wheel at the top right. (**D**) Zoomed-in fiber orientation vector map (orange box in (B)), including part of two white matter tracts (arrows) with distinct fiber orientations. Vectors of 15x15 pixels are overlaid. (**E**) Human hippocampal body, photograph of the frozen section. (**F**) ComSLI average scattering map, 8μm/pixel, including the Cornu Ammonis subfields (bright) and surrounding white matter (dark). (**G**) Corresponding fiber orientation map. (**H**) Zoomed-in fiber orientation vector map (orange box in (F)), depicting the convoluted tracts of the hippocampus, including the alveus (al), the parahippocampal gyrus white matter (phg wm), and the perforant pathway (p). Vectors of 15x15 pixels are overlaid.

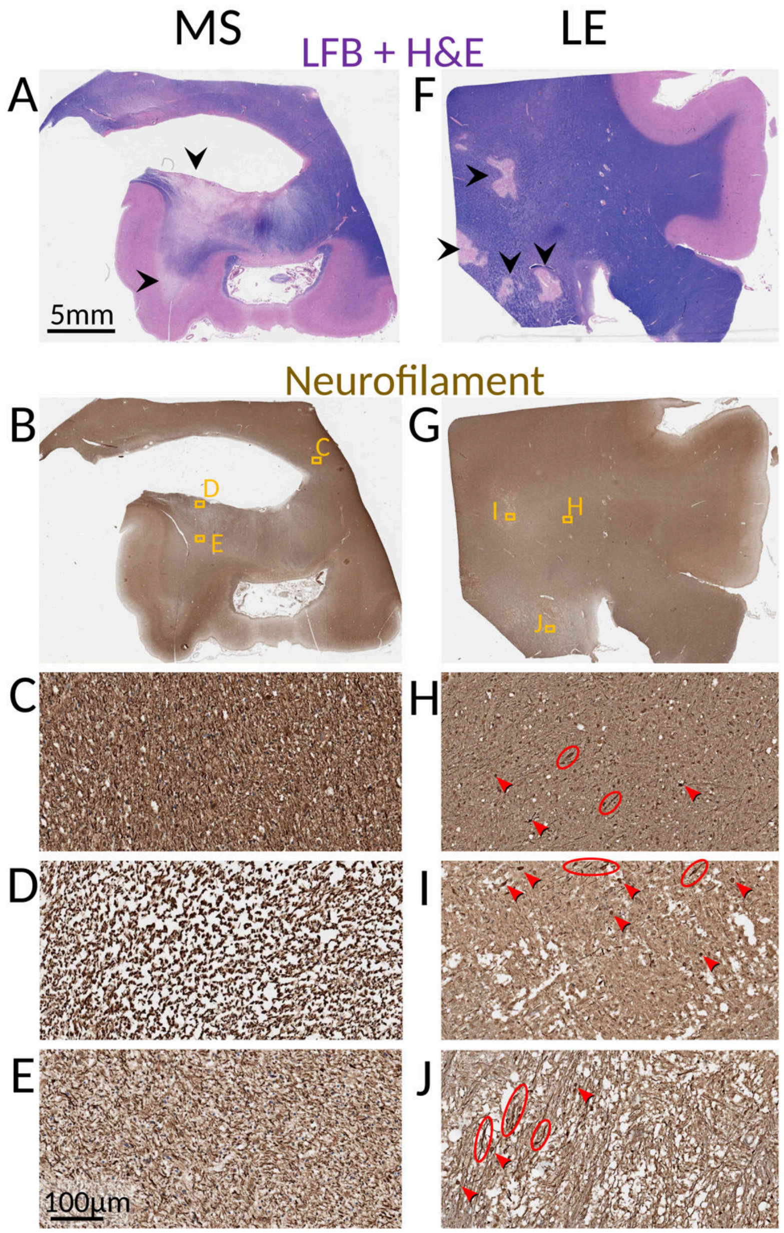

Supplementary Fig. 6 | Multiple sclerosis (MS) and leukoencephalopathy (LE) brain samples from main Fig. 3 (LFB+H&E and neurofilament-stained). (**A**) Multiple sclerosis brain section with Luxol-fast-blue (LFB) and hematoxylin & eosin (H&E) stain; LFB stains myelin blue, showing the demyelinating gradient, with minimal myelin left near the ventricle and the cortex (arrowheads). (**B**) Consecutive neurofilament-stained section, with orange boxes zoomed-in in C-E. (**C**) Normal-appearing white matter with neurofilament stain, with high density of neurofilament stain and healthy cell nuclei. (**D**) High myelin loss region, with oblique axons and tissue separation leading to low ComSLI signal in main Fig. 3B. (**E**) Region of lower myelin loss, with preserved axons, lower stain and nuclei density, leading to a bit lower scattering signal only in main Fig. 3B. (**F**) Leukoencephalopathy brain section LFB+H&E stained, with distinct fully demyelinated lesions (black arrowheads). (**G**) Consecutive neurofilament-stained section, with orange boxes zoomed-in in H-J. Examples of axonal spheroids and thickened dystrophic axons are indicated by red arrowheads and ellipsoids, respectively. (**H**) Relatively normal-appearing white matter with neurofilament stain, with high density of neurofilament stain and healthy cell nuclei, with only occasional axonal spheroids and dystrophic axons. (**I, J**) More involved areas of partial spongiosis and disrupted axons, with region-wise sparser stain and lower nuclei density.

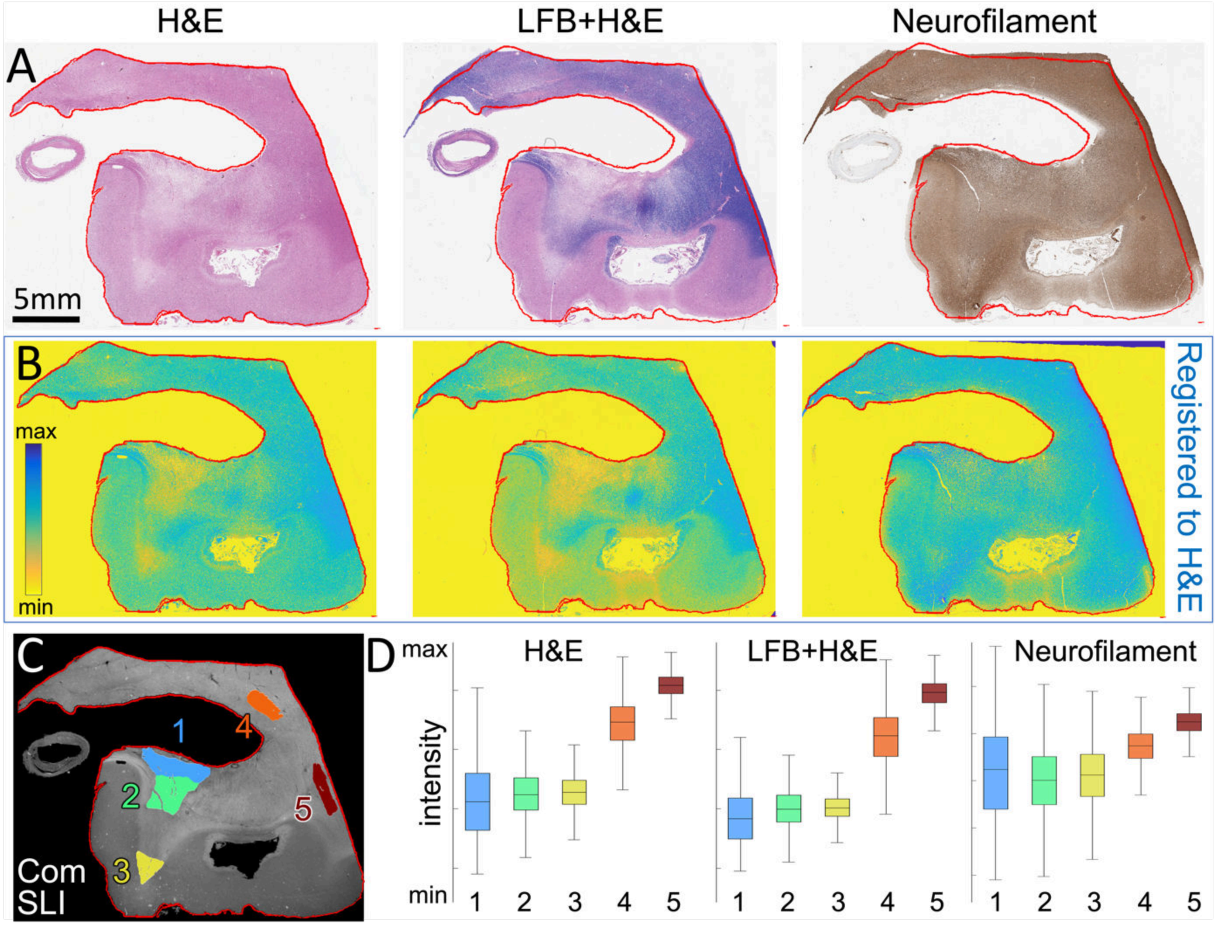

Supplementary Fig. 7 | Consecutive histology staining of human brain section with multiple sclerosis. **(A)** Brightfield images of the hematoxylin and eosin (H&E), Luxol fast blue (LFB), and neurofilament-stained consecutive sections, with a red contour indicating the outline of the H&E section which is slightly different from the LFB and neurofilament section contours. (**B**) Color intensity maps of the three sections after being registered to the H&E section, with the contour accurately matching each section contour. The registration enables quantification of the stain intensities in the lesion and normal white matter areas as annotated in main Fig. 3A. (**C**) Average scattering signal from a ComSLI measurement of the H&E stained brain section. Colored regions mark lesion areas 1-3 and normal white matter areas 4-5, the same areas indicated in main text Fig. 3. (**D**) Box plots showing the average stain intensity signal in the different areas. The signal quantification shows a clear differentiation of demyelinated areas (1-3) compared to normal myelinated areas (4-5) by H&E and LFB+H&E, with very similar values, and a less clear differentiation using neurofilament stain intensity. Box plots were generated from all pixels contained in the respective regions [1,2,3,4,5], n=[1.33, 1.35, 0.56, 0.66, 0.68]*10^5 pixels. H&E box plot: range=[0-157, 14-121, 29-109, 71-183, 119-186], quartiles=[37-85, 54-81, 59-79, 113-141, 144-161], median=[61, 67, 69, 128, 153] intensity units. LFB+H&E box plot: range=[2-115, 10-100, 27-85, 50-182, 108-186], quartiles=[27-64, 44-67, 49-63, 99-132, 137-157], median=[47, 55, 56, 117, 148] intensity units. Neurofilament box plot: range=[0-248, 7-206, 31-199, 85-195, 125-199], quartiles=[73-151, 82-131, 94-136, 126-154, 153-171], median=[115, 107, 115, 140, 162] intensity units. Source data are provided as Source Data file.

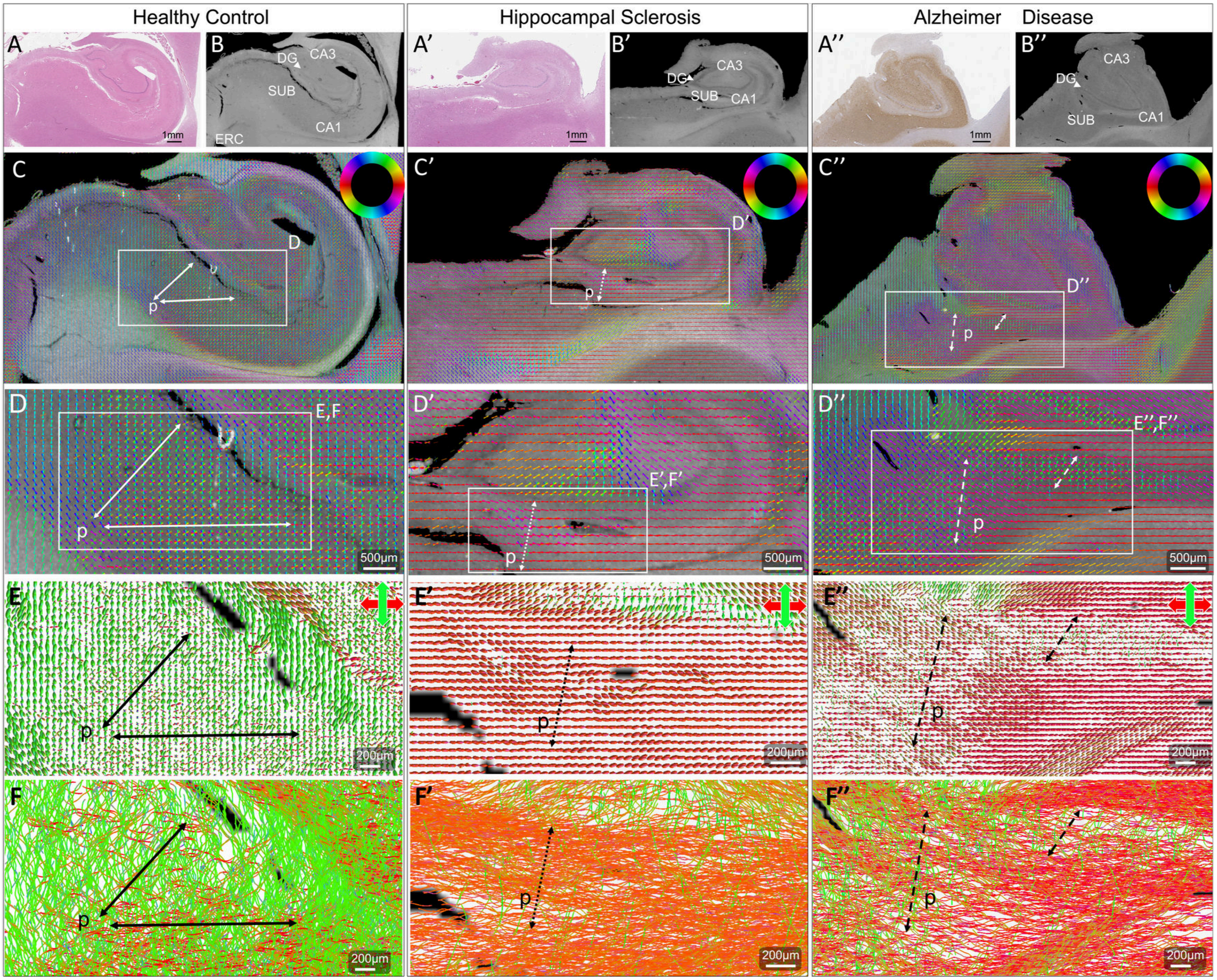

Supplementary Fig. 8 | Degenerative changes in human hippocampal tracts visualized with ComSLI. Left: healthy hippocampus (H&E-stained FFPE section). Middle: hippocampus with epileptic hippocampal sclerosis (H&E-stained FFPE section). Right: hippocampus with Alzheimer disease (tau-stained FFPE section). (**A**) Brightfield microscopy images, 0.5µm/pixel. (**B**) ComSLI average scattering maps, 7µm/pixel, with hippocampal subfields identified (SUB: subiculum, DG: dentate gyrus, CA1/3: cornu ammonis 1/3, ERC: entorhinal cortex). Note that tissue tears are present in the subiculum in both control and sclerotic specimens. (**C**) Fiber orientations, overlaid for sets of 15x15 pixels, with orientations encoded by the orientation of the lines and their color, corresponding to the color wheel in the top right. Perforant pathway (p) directions are indicated by arrows. (**D**) Zoomed-in fiber orientation vector maps (white boxes in C), including the perforant pathway crossing the subiculum and/or CA1. Vectors of 15x15 pixels are overlaid. (**E**) Orientation distribution functions (ODFs) representing fiber orientations at 50µm resolution, with perforant tract directions highlighted. (**F**) Tractograms derived from the ODFs. The control perforant pathway shows strong connections (black arrows), whereas the perforant pathway tracts in the sclerotic hippocampus (F') are very scarce, and significantly reduced in the Alzheimer Disease hippocampus (F").

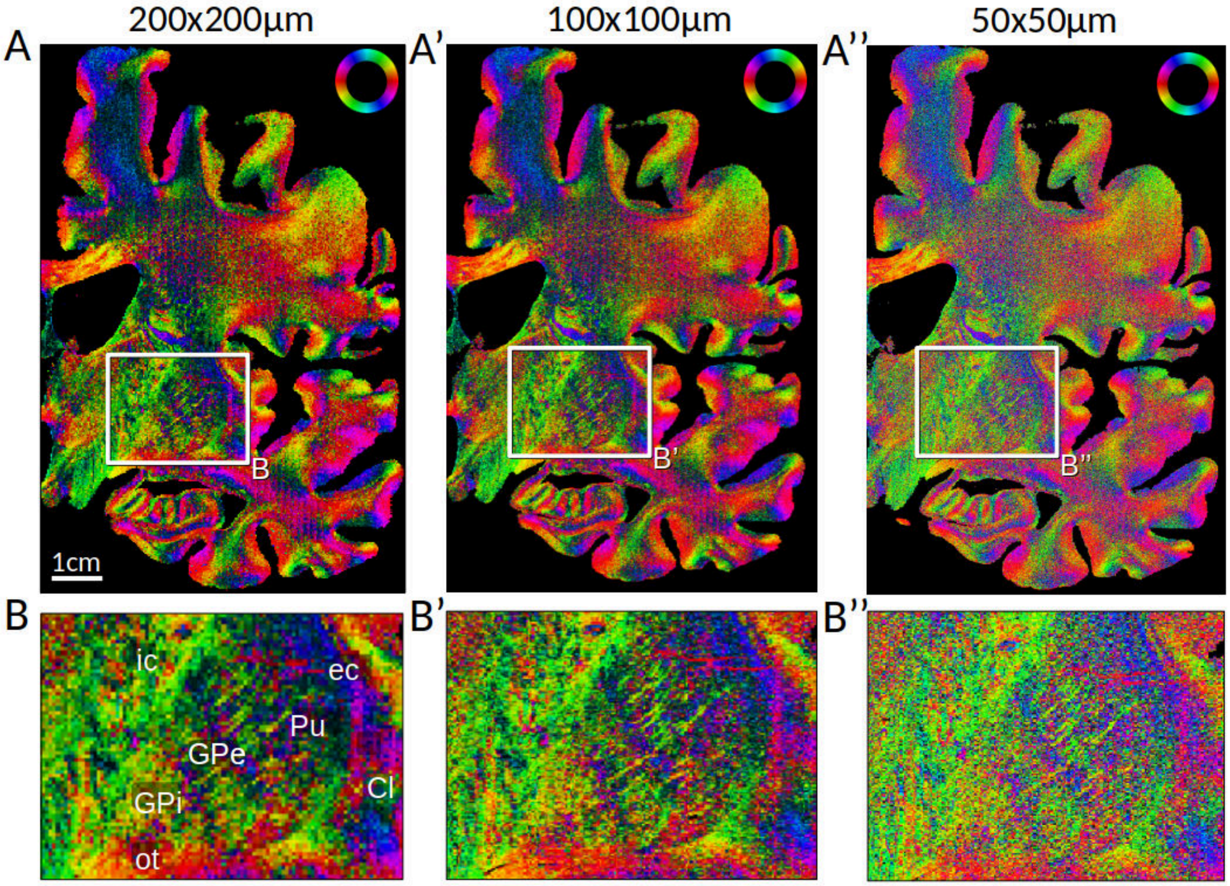

Supplementary Fig. 9 | Nissl-ST fiber orientations computed for different kernel sizes. (**A**) Fiber orientations of the Cresyl violet-stained brain section no. 3301 from main Fig. 4A computed from the brightfield microscopy image (1µm/pixel) with the provided code for Nissl-ST[2], using 15µm blur radius and a kernel size of 200x200µm, 100x100µm, and 50x50µm. (**B**) Zoom-in of the rectangular areas marked in (A), with anatomical regions labeled as in main Fig. 4B.

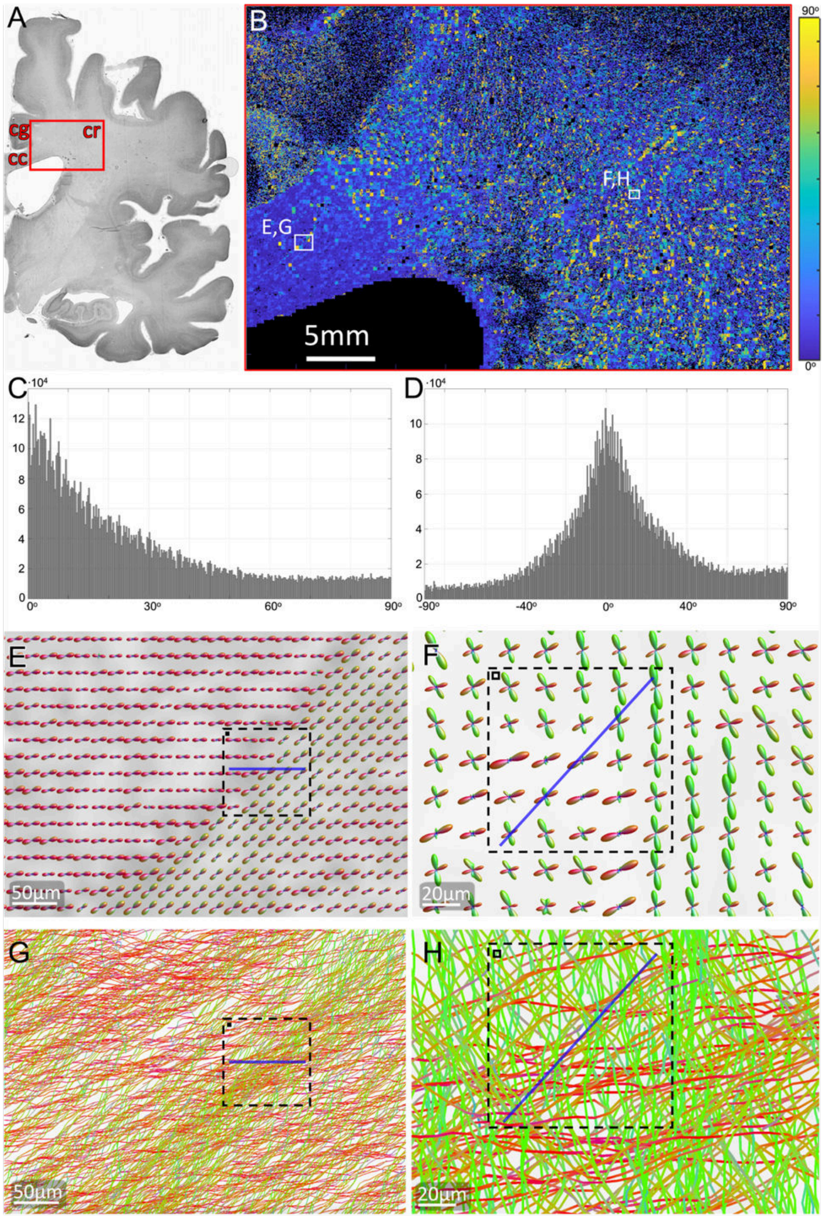

Supplementary Fig. 10 | Difference between ComSLI and Nissl-ST fiber orientations. (**A**) Histology image of the Cresyl violet-stained brain section no. 3301 from main Fig. 4A (in black-and-white), with the analyzed area marked by a red rectangle, containing part of the corpus callosum (cc), cingulum (cg), and corona radiata (cr). (**B**) Absolute angle difference map between ComSLI and Nissl-ST fiber orientations. The map shows high agreement in the corpus callosum area, with only a few Nissl-ST pixels misaligned, but a wide range of angle differences in the corona radiata, as also exemplified in main Fig. 4D,D', where Nissl-ST misidentifies the single fiber orientation angles due to the existence of abundant crossing fibers. (**C**) Histogram of absolute angle differences (10 329 010 pixels) with 0.3° bin size, showing a skewed distribution towards 0° but also significant populations of orientation differences across the angular spectrum. (**D**) Histogram of signed angle differences (ComSLI – Nissl-ST) with 0.5° bin size, showing a similar pattern as C, centered around 0°. (**E-F**) ComSLI-derived orientation distribution functions (ODFs) of the corpus callosum and corona radiata areas indicated by white boxes in B, computed for kernels of 7x7 pixels (~20μm). The size of a Nissl-ST pixel (100μm) is indicated by a dashed square, whereas the size of a ComSLI pixel is indicated by a solid square at the top left corner of the dashed square. The derived Nissl-ST orientation for the particular pixel is shown with a blue line. (**G-H**) Tractograms of the same areas as in (E-F) computed from the ComSLI-derived ODF map, with fiber diameters set to approximately 1μm. Nissl-ST and ComSLI pixel sizes are indicated by dashed and solid squares as in (E-F), and Nissl-ST single pixel orientations are shown as blue lines. Source data for the histograms in C and D are provided as Source Data file.

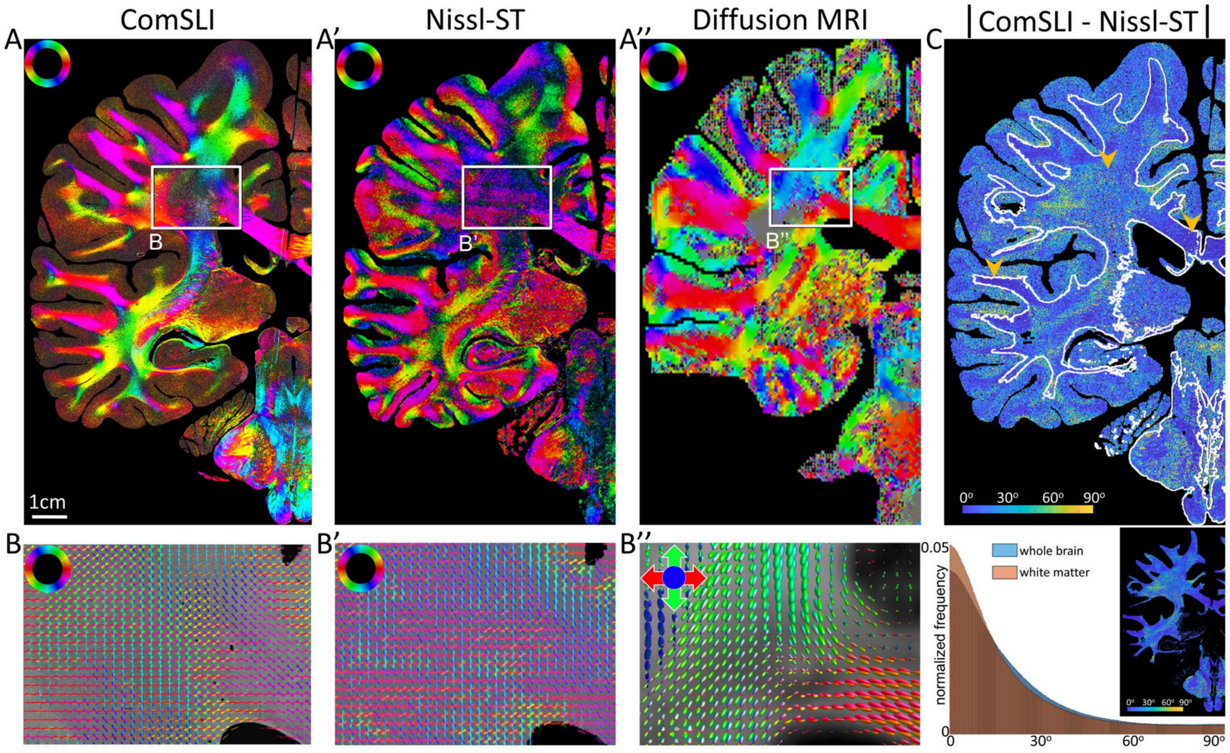

Supplementary Fig. 11 | Comparison of ComSLI, Nissl-ST, and dMRI fiber orientations for a silver-stained human brain section. ComSLI and Nissl-ST were performed on the same brain section (second *BigBrain* dataset, section no. 3452, FFPE, silver-stained, cf. main Fig. 1) – the ComSLI measurement with 9µm pixel size, and Nissl-ST on the brightfield microscopy image with 1µm pixel size and 100µm kernel size. Diffusion MRI was performed *in-vivo* on a healthy volunteer with 0.76mm isotropic voxel size, and the orientation distribution functions were evaluated at a plane similar to the evaluated brain section (see Methods). (**A**) Color-coded fiber orientations depicted for each image pixel. The orientations were computed with *SLIX*[3] for ComSLI and dMRI, and with the provided code for Nissl-ST[2], using 15µm blur radius and 100x100µm kernel. (**B**) Enlarged views for the rectangular area marked in (A), featuring crossing fibers in the corona radiata. ComSLI fiber orientations were visualized as colored lines and overlaid on 200x200 pixels for better visualization and comparison; Nissl-ST orientations were overlaid on 5x5 pixels; dMRI orientation distribution functions (ODFs) were visualized with *MRtrix3*'s *mrview*[4]. (**C**) Absolute difference between fiber orientations from ComSLI and Nissl-ST. The top image shows the difference map for the whole brain section (hemisphere), with arrows showing example white matter areas of mostly unidirectional fibers (left: superior temporal lobe; right: corpus callosum) where agreement is high, and crossing fibers (middle: corona radiata) where agreement is lower. The bottom histograms show the distribution of the difference values for the whole brain section (35 190 456 pixels, 0.2° bin size) as well as for white matter regions (20 046 296 pixels, 0.3° bin size) as indicated by the white outlines in the top image. The histograms were normalized as probability density function. Source data for the histograms in C are provided as Source Data file.

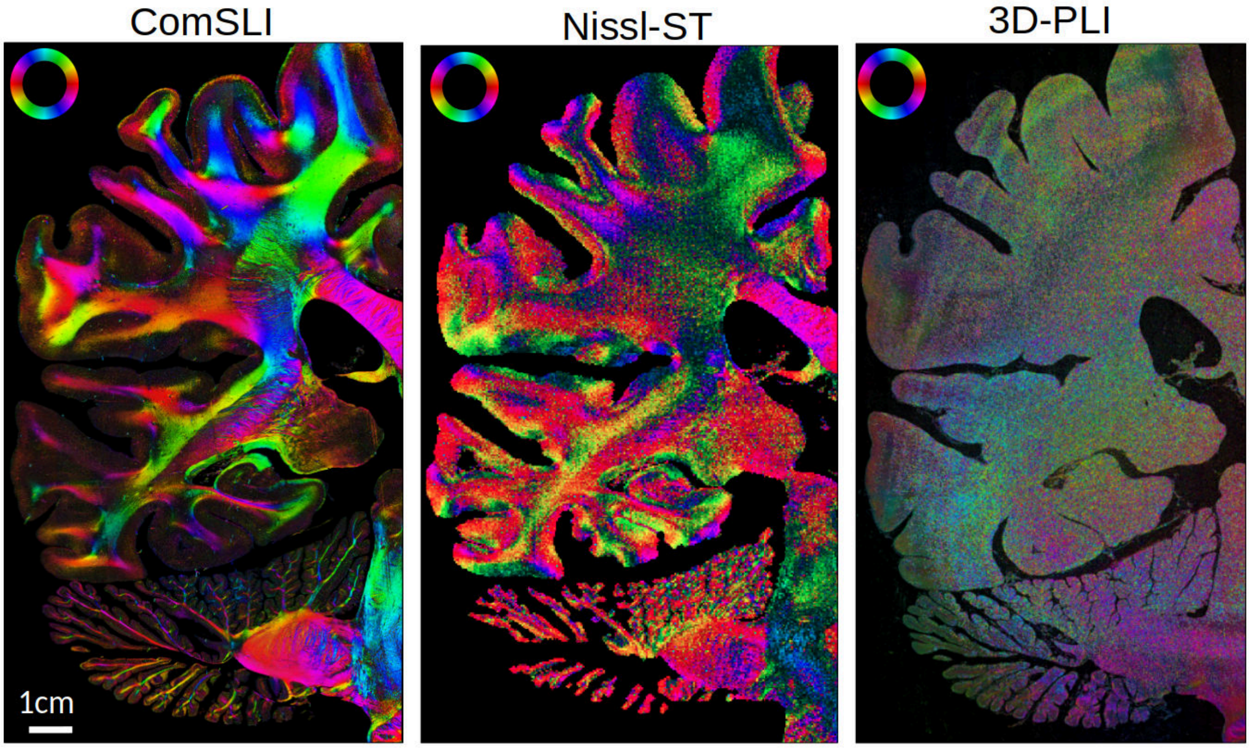

Supplementary Fig. 12 | 3D-PLI fiber orientation map of an FFPE brain section in comparison to those obtained by ComSLI and Nissl-ST. One hemisphere of a Cresyl violet-stained, human coronal brain section (no. 2520) was measured with 3D-PLI (1.85µm/pixel) and ComSLI (3µm/pixel), and the fiber orientations were computed for comparison. Due to brain preparation, the organization of myelin was destroyed and led to the loss of birefringence, going along with an impairment of fiber contrast in 3D-PLI measurements. The Nissl-ST fiber orientations were computed from the brightfield microscopy image (1µm/pixel) with 15µm blur radius and 100x100µm kernel size.

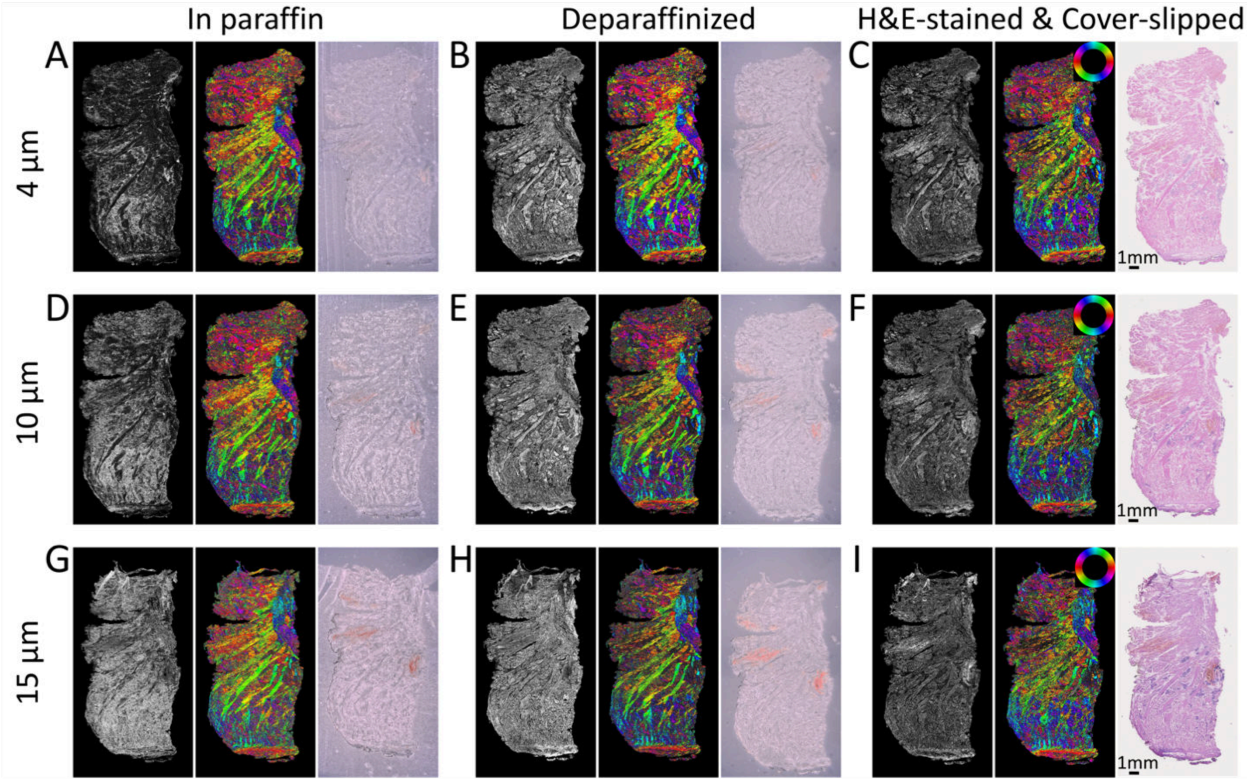

Supplementary Fig. 13 | Human tongue sections of 4µm, 10µm, and 15µm thickness measured with ComSLI at different steps of sample preparation. Each row corresponds to three different sample preparation steps of a single section of the indicated thickness. The left and middle images of each panel show the average scattering signal and the color-coded fiber orientations from ComSLI respectively (3µm/pixel), the right images show the corresponding brightfield microscopy images (0.46µm/pixel for H&E-stained sections, 10µm/pixel for the other sections). Panels in the left column (A,D,G) show the sections still in paraffin, unstained and without coverslip. Panels in the middle column (B,E,H) show the same sections after deparaffinization. Panels in the right column (C,F,I) show these sections after staining with hematoxylin and eosin (H&E) and coverslipping.

Supplementary References

1. Zeineh, M. M. *et al.* Direct Visualization and Mapping of the Spatial Course of Fiber Tracts at Microscopic Resolution in the Human Hippocampus. *Cereb Cortex* **27**, 1779–1794 (2017).
2. Schurr, R. & Mezer, A. A. The glial framework reveals white matter fiber architecture in human and primate brains. *Science* **374**, 762–767 (2021).
3. Reuter, J. A. & Menzel, M. SLIX: A Python package for fully automated evaluation of Scattered Light Imaging measurements on brain tissue. *J. Open Source Softw.* **5**, 2675–2675 (2020).
4. Tournier, J.-D. *et al.* MRtrix3: A fast, flexible and open software framework for medical image processing and visualisation. *NeuroImage* **202**, 116137 (2019).
