# Supplementary material for: Micron-resolution fiber mapping in histology independent of sample preparation
Source: Nat Commun. 2025 Nov 5;16:9572. doi: 10.1038/s41467-025-64896-9 (PMC12589536; doi:10.1038/s41467-025-64896-9)
Supplement: Supplementary file 2 — Reporting Summary [file 41467_2025_64896_MOESM2_ESM.pdf]

nature portfolio

Corresponding author(s): Marios Georgiadis, Miriam Menzel

Last updated by author(s): Aug 20, 2025

Reporting Summary

Nature Portfolio wishes to improve the reproducibility of the work that we publish. This form provides structure for consistency and transparency in reporting. For further information on Nature Portfolio policies, see our Editorial Policies and the Editorial Policy Checklist.

Statistics

For all statistical analyses, confirm that the following items are present in the figure legend, table legend, main text, or Methods section.

n/a	Confirmed	
☐	☒	The exact sample size (*n*) for each experimental group/condition, given as a discrete number and unit of measurement
☐	☒	A statement on whether measurements were taken from distinct samples or whether the same sample was measured repeatedly
☒	☐	The statistical test(s) used AND whether they are one- or two-sided<br>*Only common tests should be described solely by name; describe more complex techniques in the Methods section.*
☒	☐	A description of all covariates tested
☒	☐	A description of any assumptions or corrections, such as tests of normality and adjustment for multiple comparisons
☐	☒	A full description of the statistical parameters including central tendency (e.g. means) or other basic estimates (e.g. regression coefficient) AND variation (e.g. standard deviation) or associated estimates of uncertainty (e.g. confidence intervals)
☒	☐	For null hypothesis testing, the test statistic (e.g. *F*, *t*, *r*) with confidence intervals, effect sizes, degrees of freedom and *P* value noted<br>*Give P values as exact values whenever suitable.*
☒	☐	For Bayesian analysis, information on the choice of priors and Markov chain Monte Carlo settings
☒	☐	For hierarchical and complex designs, identification of the appropriate level for tests and full reporting of outcomes
☒	☐	Estimates of effect sizes (e.g. Cohen's *d*, Pearson's *r*), indicating how they were calculated

Our web collection on statistics for biologists contains articles on many of the points above.

Software and code

Policy information about availability of computer code

Data collection	No specific software was used for data collection.
Data analysis	The initial flat-field correction of raw ComSLI images was performed with custom MATLAB code as described in the respective section. The ComSLI fiber orientation maps were generated with the open-source software SLIX, version 2.4.2, available on GitHub (https://github.com/3d-pli/SLIX/). The Nissl-ST fiber orientation maps were generated in MATLAB using code shared by Shurr & Mezer (Science 374, 762-767, 2021). The MRI orientation distribution functions (ODFs) were computed with the open-source software MRtrix3 which is available on GitHub (https://github.com/MRtrix3/) and visualized with the tool mrview. The MATLAB code to generate µFODs from ComSLI fiber orientation maps, create a virtual diffusion MRI dataset, and generate ODFs and tractography using MR tools is provided in the Dryad repository (https://doi.org/10.5061/dryad.02v6wwqb2).

For manuscripts utilizing custom algorithms or software that are central to the research but not yet described in published literature, software must be made available to editors and reviewers. We strongly encourage code deposition in a community repository (e.g. GitHub). See the Nature Portfolio guidelines for submitting code & software for further information.

Data

Policy information about availability of data

All manuscripts must include a data availability statement. This statement should provide the following information, where applicable:

- Accession codes, unique identifiers, or web links for publicly available datasets
- A description of any restrictions on data availability
- For clinical datasets or third party data, please ensure that the statement adheres to our policy

All data underlying the study, the original image files, and a sample whole-brain dataset are available in the data repository Dryad: https://doi.org/10.5061/dryad.02v6wwqb2.

Research involving human participants, their data, or biological material

Policy information about studies with human participants or human data. See also policy information about sex, gender (identity/presentation), and sexual orientation and race, ethnicity and racism.

Reporting on sex and gender	Most human brain samples were acquired from male body donors; the Luxol-fast-blue stained sample and the sclerotic and control hippocampus samples were acquired from female body donors. Human non-brain tissues (tongue, colorectal, bone, and artery wall) were obtained from a tissue archive at Erasmus Medical Center in Rotterdam (the Netherlands); sex or gender of these patients is unknown. As the focus of the study is to reconstruct intricate nerve fiber architectures in various archived brain tissue sections, sex or gender did not play a role in this study.
Reporting on race, ethnicity, or other socially relevant groupings	Race, ethnicity, or other socially relevant groupings are unknown and were not relevant in this study.
Population characteristics	The whole human brain sections were obtained from 25-, 30- and 71-year-old body donors without neurological disorders. The hippocampal sections were from an 89-year-old body donor with Alzheimer pathology. Samples with multiple sclerosis were from an 80-year-old body donor. Samples with leukoencephalopathy were from a 43-year-old body donor. Hippocampal and visual cortex sections were from a 60- and 67-year-old body donor. The sclerotic hippocampal section was from a 69-year-old body donor with epilepsy, the control from a 66-year-old donor with no neuropathologic abnormality. The human hippocampus and primary visual cortex fresh-frozen sections were from an 88-year-old male with Lewy Body Disease, low Alzheimer’s disease pathology, and cerebrovascular dementia.
Recruitment	For this study, only archived tissue sections were used. No participants were recruited for the study.
Ethics oversight	Our research complies with all relevant ethical regulations. The body donors gave written informed consent for the general use of postmortem tissue used in this study for aims of research and education. The whole human brain sections were acquired in accordance with the ethics committee of the medical faculty of the Heinrich Heine University Düsseldorf (Germany) under protocol no. 4863. All other human brain samples were acquired in accordance with the Stanford Alzheimer’s Disease Research Center (ADRC) Institutional Review Board (IRB), Assurance no. FWA00000935. The usage of the non-brain tissue samples was approved by the Medisch Ethische Toetsing Commissie (METC) of the Erasmus Medical Center in Rotterdam (the Netherlands) under project no. MEC-2023-0587.

Note that full information on the approval of the study protocol must also be provided in the manuscript.

Field-specific reporting

Please select the one below that is the best fit for your research. If you are not sure, read the appropriate sections before making your selection.

[x] Life sciences   [ ] Behavioural & social sciences   [ ] Ecological, evolutionary & environmental sciences

For a reference copy of the document with all sections, see nature.com/documents/nr-reporting-summary-flat.pdf

Life sciences study design

All studies must disclose on these points even when the disclosure is negative.

Sample size	No statistical method was used to predetermine sample size; no sample size calculation was performed. We have chosen a representative set of samples (various types of histological tissue sections) to demonstrate the capabilities of our technique to map fibers in various tissue samples. We show measurement results on more than 35 different tissue sections (adjacent tissue sections, different tissue types, tissues from different species, tissues with different pathologies, tissues with different thicknesses, differently stained tissues, differently prepared tissues). Sample sizes were chosen such that they support our hypothesis (that ComSLI can map fibers in histological tissue sections independent of sample preparation). The applicability of our technique was demonstrated on brain tissue sections obtained from 1 mouse, 1 pig, and 11 human body donors (including one with Alzheimer, one with multiple-sclerosis brain, one with leukoencephalopathy, one with epilepsy, and one with Lewy Body Disease, low Alzheimer's disease pathology, and cerebrovascular dementia). In addition, we performed measurements on non-brain tissues (tongue muscle sections, colorectal tissue sections, bone sections, and artery wall sections) which were obtained from a tissue archive where patients are unknown.
Data exclusions	No data were excluded from the analysis.

Replication	Measurements were mostly taken from distinct samples. The reproducibility of the experimental findings was demonstrated, e.g., by performing measurements of the same sample after different steps of sample preparation or by performing measurements across different species. To show that our technique can reconstruct fiber organization independent of sample preparation, we have also measured several adjacent tissue sections with different staining and different tissue thicknesses. All these attempts of replication were successful and confirm that our results are reproducible.<br><br>Control of covariates was not applicable in our study; the aim was to demonstrate the capabilities of our technique (micron-resolution fiber mapping across various histological tissue samples) and not how variables might affect measurement outcomes.
Randomization	The experiments were not randomized; the studies were performed on existing archived tissue sections. To show the broad applicability of our technique, we selected various samples from different species (mouse, pig, human) and different tissue types (brain, muscle, bone, artery). In addition, we studied healthy and neuropathological tissues, including multiple sclerosis and leukoencephalopathy.
Blinding	The Investigators were not blinded to allocation during experiments and outcome assessment. Our study shows the capabilities of ComSLI to map fibers in various histological tissue samples; the same measurement and analysis procedures were applied to all samples independent of their origin or preparation. Blind studies were therefore not necessary for demonstrating the capabilities of our method.

Reporting for specific materials, systems and methods

We require information from authors about some types of materials, experimental systems and methods used in many studies. Here, indicate whether each material, system or method listed is relevant to your study. If you are not sure if a list item applies to your research, read the appropriate section before selecting a response.

Materials & experimental systems

n/a	Involved in the study	
☐	☒	Antibodies
☒	☐	Eukaryotic cell lines
☒	☐	Palaeontology and archaeology
☐	☒	Animals and other organisms
☒	☐	Clinical data
☒	☐	Dual use research of concern
☒	☐	Plants

Methods

n/a	Involved in the study	
☒	☐	ChIP-seq
☒	☐	Flow cytometry
☐	☒	MRI-based neuroimaging

Antibodies

Antibodies used	Antibodies used in Alzheimer Disease brain tissue were previously reported in Zeineh et al., Neurobiol Aging 36(9), 2483-2500 (2015):<br>- Tau: Phospho-tau-AT8 anti-human paired helical filaments-tau monoclonal antibody (10 µg/mL, MN1020, Pierce Biotechnology, Rockford, IL 61105, USA).<br>- Microglia: CD163 antibody (NCL-CD163, Leica Biosystems Newcastle Ltd, Newcastle Upon Tyne, UK)<br>- Amyloid: monoclonal mouse anti-human Aβ antibody (M0872, Dako Canada, Inc, Burlington, ON, USA)<br>The mouse brain section was incubated with anti-Iba1 rabbit monoclonal antibodies (abcam, Cambridge, MA).
Validation	The primary antibodies were previously validated in other research studies (Zeineh et al., Neurobiol Aging 36(9), 2483-2500, 2015).

Animals and other research organisms

Policy information about studies involving animals; ARRIVE guidelines recommended for reporting animal research, and Sex and Gender in Research

Laboratory animals	one 10-week-old C57BL/6 female mouse (Jackson Laboratories), one 4-week-old Yorkshire female pig
Wild animals	The study did not involve wild animals.
Reporting on sex	The animals used in this study are female. However, sex was not considered in the study design as it is not the focus of our study. Our study focuses on the demonstration that the presented imaging technique can reliably reconstruct fiber organization in various tissues independent of sample preparation.
Field-collected samples	The study did not involve samples collected from the field.
Ethics oversight	The mouse and the pig were euthanized for the purposes of different studies, approved by the Stanford Administrative Panel on Laboratory Animal Care (APLAC). Mouse: Stanford APLAC protocol no. 32577, Pig: Stanford APLAC protocol no. 33684. The archived brain sections were reused for this study.

Note that full information on the approval of the study protocol must also be provided in the manuscript.

Plants

Seed stocks	No plants were used for this study.
Novel plant genotypes	No plants were used for this study.
Authentication	No plants were used for this study.

Magnetic resonance imaging

Experimental design

Design type	We compared our data to a publicly available in-vivo whole-brain diffusion MRI reference dataset of 760µm isotropic resolution from a ca. 30-year-old, healthy male patient:<br><br>Wang, F. et al., "In vivo human whole-brain Connectom diffusion MRI dataset at 760µm isotropic resolution", Scientific Data 8, 122 (2021).
Design specifications	No specific trial was performed.
Behavioral performance measures	No behavioral tasks were performed.

Acquisition

Imaging type(s)	diffusion MRI
Field strength	3T
Sequence & imaging parameters	(see above reference for complete information)<br><br>The dataset was acquired at 760µm isotropic resolution, and sampled at 1260 q-space points across 9 two-hour sessions, including 420 directions at b = 1000 s/mm² and 840 directions at b = 2500 s/mm² across the 9 sessions to ensure high angular resolution. The scans were performed with a MGH-USC 3T Connectom scanner with maximum gradient amplitude of 180 mT/m and a slew rate of 125 T/m/s, a custom-built 64-channel phased-array coil, a personalized motion-robust head stabilizer, an SNR-efficient simultaneous multi-slab sequence (gSlider-SMS), parallel imaging reconstruction with advanced ghost reduction algorithm (Dual-Polarity GRAPPA), and reversed phase-encoding acquisition.<br><br>A gSlider encoding factor of 5 and a multi-band acceleration factor of 2 were used to achieve high SNR efficiency by acquiring 10 slices per shot across two RF-encoded slabs, where each slab contains 5 slices that can be resolved through 5 gSlider-encoded repetitions. In-plane acceleration of 3 was selected to mitigate EPI distortion and blurring by reducing the effective echo spacing from 1.02 ms to 0.34 ms, while ensuring good reconstruction performance with low noise amplification when the reconstruction was performed with Dual polarity GRAPPA to also mitigate ghosting artifacts.<br><br>Before diffusion acquisition, a calibration scan was acquired using a multi-shot mono-polar SE-EPI to provide fully-sampled data, with the same effective echo spacing as the in-plane accelerated data and in both readout polarities, to calculate the dual-polarity GRAPPA kernel.
Area of acquisition	whole-brain

Diffusion MRI ☒ Used ☐ Not used

Parameters	(see above reference for complete information)<br><br>A total of 2808 volumes of dMRI data were acquired across the 9 sessions, consisting of 144 b = 0 s/mm² images, 420 b = 1000 s/mm² diffusion-weighted images (DWIs) and 840 b = 2500 s/mm² DWIs, along with their paired reversed phase-encoding volumes.<br><br>The image reconstruction for dMRI was performed on raw k-space data in MATLAB. First, dual-polarity-GRAPPA (DPG) was used for parallel imaging reconstruction of both in-plane and multi-band accelerations. Unaliased multi-channel images were combined using SENSE 1 complex coil combination method, where the sensitivity maps were estimated from the calibration data using ESPIRiT. POCS was used for partial Fourier reconstruction to recover high spatial information from partially acquired k-space data. Diffusion background phase-corruptions were further removed using the real-valued diffusion algorithm to provide real-value data and avoid magnitude bias in subsequent post-processing steps. Finally, gSlider's slab-to-slice reconstruction was performed to reconstruct thick RF-encoded slabs (3.8 mm) into thin slices (0.76 mm), where T1 recovery was incorporated into the reconstruction model to mitigate slab-boundary artifacts.

Preprocessing

Preprocessing software	FMRIB Software Library (FSL)
Normalization	The non-DW images (b = 0 s/mm²) were used to normalize the signal intensity of the data acquired within and across the different acquisition blocks (~25 min each). The signal intensity differences across acquisition blocks were normalized to the mean signal intensity of the first session.
Normalization template	(see above reference for details)
Noise and artifact removal	Preprocessing corrections across the reconstructed image series were performed to correct for signal drift, susceptibility, eddy currents, and gradient nonlinearity induced distortions, and participant motion.
Volume censoring	(see above reference for details)

Statistical modeling & inference

Model type and settings	(see above reference for details)
Effect(s) tested	(see above reference for details)

Specify type of analysis: ☒ Whole brain ☐ ROI-based ☐ Both

Statistic type for inference<br>(See Eklund et al. 2016)	(see above reference for details)
Correction	(see above reference for details)

Models & analysis

n/a	Involved in the study
☒	☐ Functional and/or effective connectivity
☒	☐ Graph analysis
☒	☐ Multivariate modeling or predictive analysis
