# Supplementary material for: Micron-resolution fiber mapping in histology independent of sample preparation
Source: Nat Commun. 2025 Nov 5;16:9572. doi: 10.1038/s41467-025-64896-9 (PMC12589536; doi:10.1038/s41467-025-64896-9)
Supplement: Supplementary file 3 — Description of Additional Supplementary Files [file 41467_2025_64896_MOESM3_ESM.pdf]

Description of Additional Supplementary Files

Supplementary Movie 1

ComSLI dataset of whole human brain BigBrain section (second BigBrain dataset, section no. 3452, FFPE, silver-stained, fiber orientations in main Fig. 1). Each frame shows pixel intensities after calibration (see Methods) for the entire brain at each illumination angle φ (in degrees). The movie, which has been 5x downsampled to reduce its size of many gigabytes, can be downloaded from the data repository Dryad: https://doi.org/10.5061/dryad.02v6wwqb2 [SupplementaryMovie1.gif].
