# Supplementary figures and images for: Micron-resolution fiber mapping in histology independent of sample preparation
Source: Nat Commun. 2025 Nov 5;16:9572. doi: 10.1038/s41467-025-64896-9 (PMC12589536; doi:10.1038/s41467-025-64896-9)

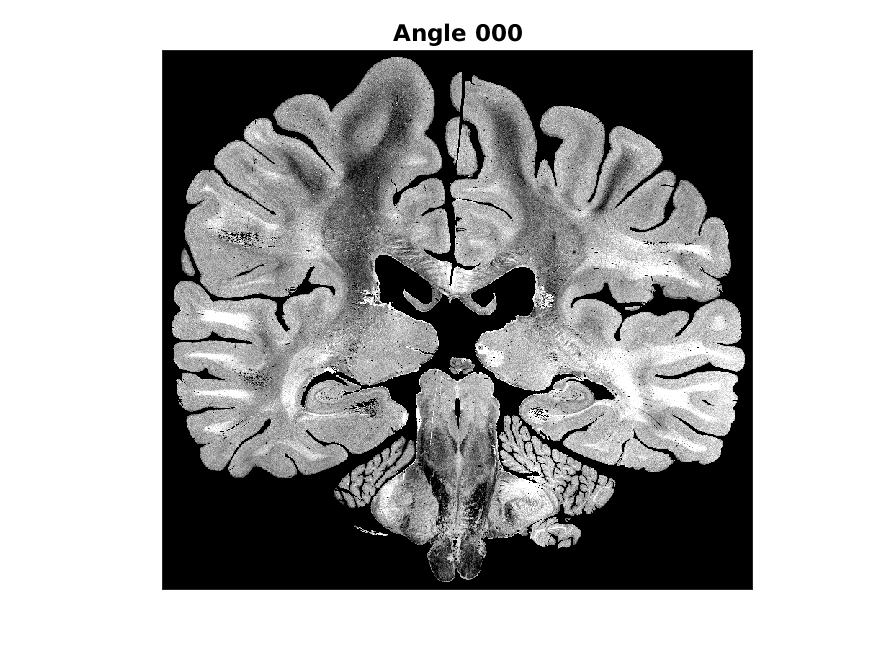

Supplement: Supplementary file 4 — Supplementary Movie 1 [file 41467_2025_64896_MOESM4_ESM.gif]
